# Enhancing the stability and porosity of penetrated metal–organic frameworks through the insertion of coordination sites†
†Electronic supplementary information (ESI) available: Experimental section, crystallographic tables, IR, TG and characterization details. CCDC 1576271 and 1576272. For ESI and crystallographic data in CIF or other electronic format see DOI: 10.1039/c7sc04192f


**DOI:** 10.1039/c7sc04192f

**Published:** 2017-11-15

**Authors:** Rui Feng, Yan-Yuan Jia, Zhao-Yang Li, Ze Chang, Xian-He Bu

**Affiliations:** a State Key Laboratory of Elemento-Organic Chemistry , College of Chemistry , Collaborative Innovation Center of Chemical Science and Engineering (Tianjin) , Nankai University , Tianjin 300071 , China . Email: buxh@nankai.edu.cn ; Fax: +86-22-23502458; b School of Materials Science and Engineering , National Institute for Advanced Materials , Tianjin Key Laboratory of Metal and Molecule-Based Material Chemistry , Nankai University , Tianjin 300350 , China

## Abstract

Guided by the insertion of coordination sites within ligands, an interpenetrated metal–organic framework NKU-**112** and a self-penetrated framework NKU-**113** were obtained. The two MOFs have similar cage-based framework structures, while NKU-**113** reveals enhanced porosity and stability compared with NKU-**112**, owing to the self-penetrated structure induced by the additional chelating bipyridine moiety in the ligand. To the best of our knowledge, this is the first study that attempts to shift the structure topology of a MOF from interpenetrated to self-penetrated while achieving a delicate modulation of the location and distances within the penetrated structure by inserting new coordination sites.

## Abstract 2

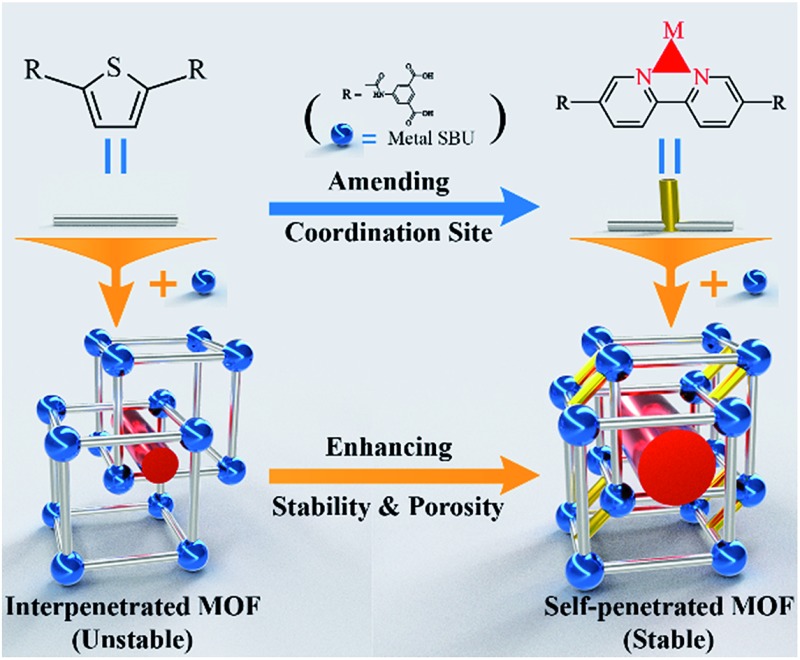
Guided by the insertion of coordination sites within ligands, an interpenetrated metal–organic framework (MOFs) NKU-**112** and a self-penetrated framework NKU-**113** were obtained. The enhanced stability and porosity of NKU-**113** prove the efficiency of the method for the structure and properties modulation of penetrated MOFs.

## Introduction

Metal–organic frameworks (MOFs) composed of metal ions/clusters connected by organic linkers have emerged as a class of attractive porous materials.^1^ Owing to their tailorable porous structures, MOFs have shown great potential in various applications such as gas storage and/or separation, catalysis, sensing, *etc.*^2^ Though significant progress has been made in the structure and property modulation of MOFs, the practical applications of MOFs have been limited by their relatively low stability.^3^ Although several strategies have been proposed to enhance the stability of MOFs, including ligand or metal ion changes, surface modification, interpenetration and the construction of multi-walls,^4^ the development of a facile and straightforward design and construction strategy for stable MOFs is still a desired research goal.^5^

Framework interpenetration frequently occurs in MOF structures, particularly when extended organic ligands are used for MOF construction. In spite of the reduction of the pore volume of the framework, framework interpenetration in MOFs has been observed to not only enhance MOF stability but also regulate the pore size, thus augmenting their gas sorption properties originating from the promoted interactions between the individual networks. For example, Zhou *et al.* have compared the H_2_ sorption performances of non-interpenetrated and two-fold interpenetrated MOFs to find that framework interpenetration could benefit the stability of the framework and gas sorption at low pressure.^6^ However, the enhancement of framework stability by interpenetration is somewhat unmanageable, since the van der Waals interactions between the organic ligands of the individual networks may not be strong enough to prevent structure deformation and framework slippage in response to the removal of guest species in the framework. On the other hand, the coordination geometry of secondary building units (SBUs) composed of metal centers with coordinated solvent molecules may vary in response to the removal of solvents, which could also affect the stability of the MOFs.

Focusing on the stability enhancement of MOFs, the combination of framework penetration and the stabilization of SBUs could be a rational strategy. In principle, if the coordination sphere of the metal center is broadened and additional metal–ligand bonds are introduced to increase the ligancy of the metal cluster and to enhance the strength of the interactions between the individual networks, the resulting self-penetrated framework may become more stable than the original interpenetrated framework. However, realizing the above-mentioned scenario remains to be a challenging task, mainly because of the mismatched distance between frameworks as well as unfavorable coordination environments for additional metal ions.

**Fig. 1:**
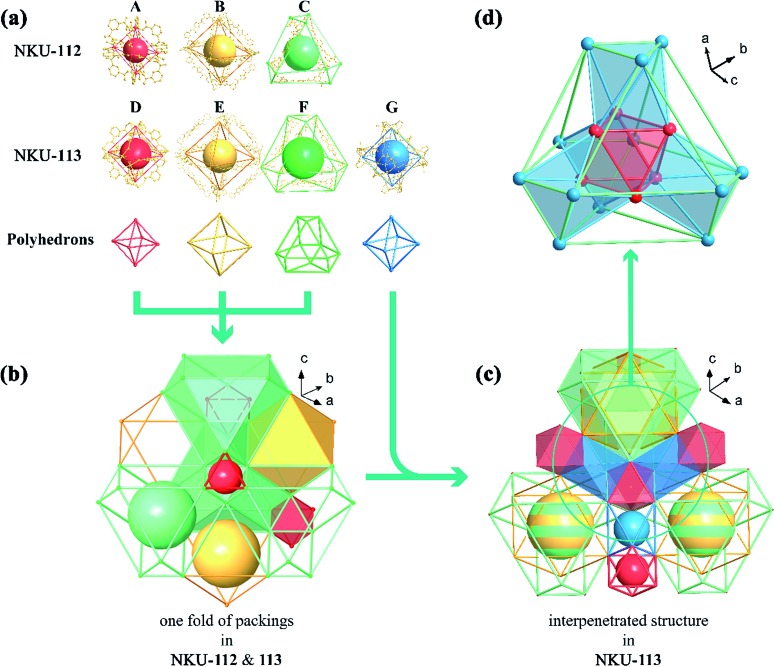
Structure diagrams of cages (a), one-fold packing cages in NKU-**112** and **113** (b), interpenetrated two-fold cages in NKU-**113** (c) and nestification relationships in NKU-**113** (d). (a) The octahedron combined with six [Ni_2_(CO_2_)_4_(μ_2_-H_2_O)(H_2_O)_2_DMF_2_] clusters and twelve isophthalic moieties in NKU-**112** (cage **A**); another octahedron combined with six Ni clusters and twelve **L1**^4–^ (cage **B**); a distorted cuboctahedron combined with six **L1**^4–^ and twelve Ni clusters (cage **C**); an octahedron combined with six [Co_2_(CO_2_)_4_(μ_2_-H_2_O)(H_2_O)_2_] clusters and twelve isophthalic acids in NKU-**113** (cage **D**); an octahedron combined with six Co clusters and twelve **L2**^4–^ (cage **E**); a distorted cuboctahedron combined with six **L2**^4–^ and twelve Co clusters (cage **F**); an octahedron combined with six Co clusters and six **L2**^4–^ (cage **G**). (b) The packing structure of the three kinds of cages in NKU-**112**. (c) The packing structure of the four kinds of cages in NKU-**113**. (d) A diagram of the nestification of cages **D**, **F**, and **G** in NKU-**113**.

**Scheme 1:**
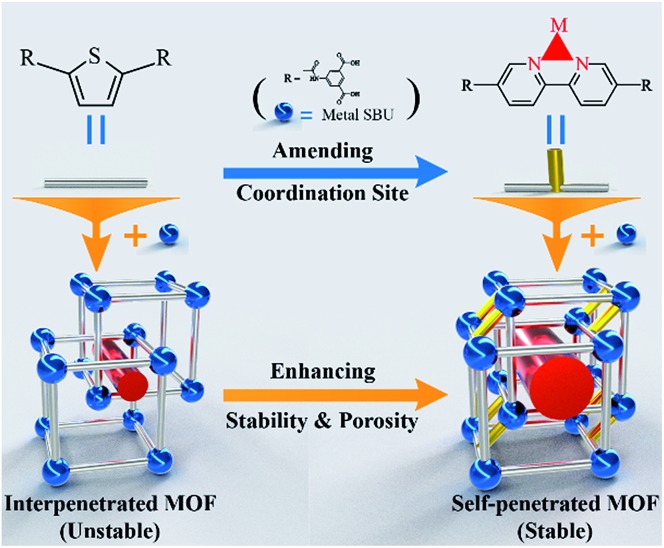
Framework and modification strategy diagram. The blue-colored balls and the silver-colored sticks represent metal secondary building units and ligands, respectively. The yellow-colored sticks represent inserted coordinate bonds, which covalently connect two sets of frameworks to each other. The red-colored cylinders represent pores, which are enlarged after ligand modification.

In this study, one such rare example is reported. By inserting additional coordinating sites (here through the introduction of bipyridine groups) in the backbone of the organic ligand, a two-fold interpenetrated framework [Ni_2_**L1**(μ_2_-H_2_O)(H_2_O)_2_(DMF)_2_]·(solvents)_*n*_ (denoted as NKU-**112**, NKU-Nankai University, DMF = *N*,*N*′-dimethylformamide, H_4_**L1** = 5,5′-((thiophene-2,5-dicarbonyl)bis(azanediyl))diisophthalic acid, shown in Fig. S1†) can be turned into a self-interpenetrated framework [Co_2_**L2**(μ_2_-H_2_O)(H_2_O)_2_]·(solvents)_*n*_ (denoted as NKU-**113**, H_4_**L2** = 5,5′-([2,2′-bipyridine]-5,5′-dicarbonyl)bis(azanediyl) diisophthalic acid, Fig. S1†), in which the extent of the mutual framework interaction is significantly increased in comparison with the two-fold interpenetrated MOF (Fig. 1). As a result, NKU-**113** displays enhanced porosity and stability with respect to NKU-**112**, although they have similar cage-based packing structures (Scheme 1). This work provides a unique strategy for the enhancement of coordination interactions for increasing the stability and porosity of penetrated MOFs.

## Results and discussion

### Structures of NKU-**112** and NKU-**113**

**Fig. 2:**
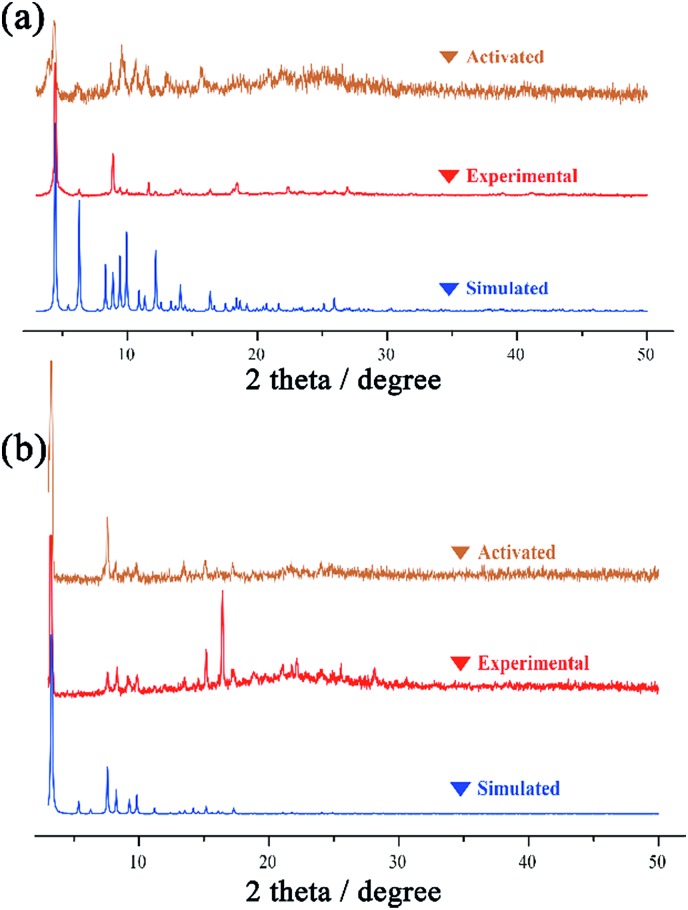
The PXRD patterns of simulated, experimental and activated samples of NKU-**112** (a) and NKU-**113** (b).

A solvothermal reaction of Ni(NO_3_)_2_·6H_2_O and H_4_**L1** in DMF and acetonitrile affords crystals of NKU-**112**, whereas crystals of NKU-**113** are obtained from the solvothermal reaction of Co(NO_3_)_2_·6H_2_O and H_4_**L2** in DMF, acetonitrile and H_2_O. The structures of NKU-**112** and NKU-**113** were determined using single-crystal X-ray diffractometry. The bulk samples of NKU-**112** and NKU-**113** were characterized using IR (Fig. S2†), and their phase purities were verified by the well matched powder X-ray diffraction (PXRD) patterns of the as-synthesized samples and the simulated ones (Fig. 2). Thermogravimetric (TG) analyses showed that NKU-**112** can retain its original structure up to a temperature of 370 °C, whereas NKU-**113** can retain its structure up to 400 °C (Fig. S3†).

**Fig. 3:**
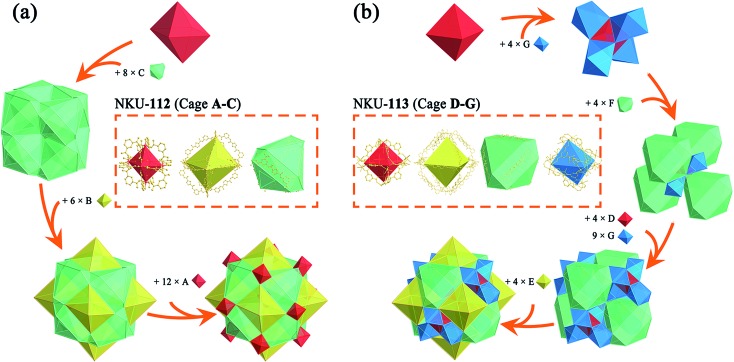
Structure combination diagrams of the cages presented in NKU-**112** (a) and NKU-**113** (b). Inset: coordination structures of cages **A–G**.

Single crystal X-ray diffraction reveals that NKU-**112** crystallises in the cubic space group *Ia*3. Apart from the guest molecules, the asymmetric unit contains two Ni^2+^ cations, one **L1**^4–^ anion, three water and two DMF molecules. The Ni1 is six coordinated in a distorted octahedron geometry with four carboxylate oxygen atoms (O1, O15, O22 and O24) from four different organic ligands, one oxygen atom (O3) from a terminal water molecule and one oxygen atom (O13) from a μ_2_-water molecule. In contrast, the coordination sphere of Ni2 includes two carboxylate oxygen atoms (O23 and O25), two oxygen atoms from terminal DMF molecules (O9 and O29), one oxygen atom from a terminal water molecule (O8) and one oxygen atom from a μ_2_-water molecule (O13), which can also be described as a distorted octahedral geometry (Fig. S4†). Ni1 and Ni2 are bridged by two carboxylate groups and one μ_2_-H_2_O molecule to form a discrete [Ni_2_(COO)_4_(μ_2_-H_2_O)(H_2_O)_2_(DMF)_2_] cluster in which a water molecule is inserted between the Ni^2+^ ions, and the Ni1–O13–Ni2 angle is close to 120° (117.463° to be precise), a completely different value to that of a classical [M_2_(COO)_4_O_2_] SBU (Fig. S5†). The SBUs were further expanded by the organic linker to form a three-dimensional (3D) network. Careful examination of the network structure reveals that the framework is composed of three different types of cage. As shown in Fig. 1, the octahedral cage **A**, with a diameter of *ca.* 6.8 Å, is defined by six Ni clusters, each being at a vertex of the octahedron, and twelve isophthalate moieties along the edges. The other octahedral cage **B**, with a diameter of *ca.* 21.6 Å, is composed of six Ni clusters and twelve organic ligands, whilst the third type of cage, the distorted cuboctahedral cage **C** with a diameter of *ca.* 15.8 Å, consists of twelve Ni clusters and six organic ligands (Fig. 1b). As for the packing of NKU-**112**, each distorted cuboctahedral **C** cage is surrounded by four **A** cages and four **B** cages. Cage **C** shares three Ni clusters and one isophthalic moiety on the truncated surface with cage **A**, and three Ni clusters and **L1**^4–^ with cage **B** (Fig. 3a). Topologically, since cage **A** occupies vertices of cage **B** and **C**, cage **A** can be regarded as nodes for clarity and the framework features a uninodal 12-connected net, which is classified as *fcu* as determined using TOPOS software (Fig. S6a†).^7^ Due to the large void in the network, two-fold interpenetration occurs in NKU-**112**, and the solvent-accessible volume of the interpenetrated NKU-**112** is estimated to be 45.7% per unit cell by PLATON.^8^

The transformation between interpenetrated and self-penetrated MOFs has been studied in the past.^9^ After specific connecting components are added, two or more folds of interpenetrated frameworks are covalently linked to each other to form a single framework. If these components are removed, the framework can be restored to its original topology. The self-penetrated structure can also be constructed using elaborate ligand design, and the key is finding a suitable ligand. Typically, as two major coordination groups, carboxylate groups and N-containing heterocyclic groups are employed in the majority of MOF constructions.^10^ Among the various groups, a chelating bipyridine moiety could form a relatively stable coordination structure with a metal center to stabilize the SBU. Accordingly, a bipyridine group was selected to be inserted in the backbone of the organic ligand H_4_**L1**, and the resulting H_4_**L2** ligand was used to construct NKU-**113**.

Single-crystal X-ray diffraction reveals that NKU-**113** crystallises in the cubic space group *Fd*3*m*. The asymmetric unit comprises two Co^2+^ cations, one **L2**^4–^ anion, and three water molecules. Co1 and Co2 are connected to each other through a bridge comprising two carboxylate groups from **L2**^4–^ and a μ_2_-water molecule. Co1 is coordinated by four **L2**^4–^ anions and two water molecules in a distorted trigonal bipyramid defined by O4, O5 and four O1 atoms from four **L2**^4–^ anions. Co2 is coordinated by two carboxyls from **L2**^4–^, two nitrogen atoms from **L2**^4–^ and two water molecules that together form another distorted trigonal bipyramid defined by two O2 atoms, two N1 atoms, O5 and O6 (Fig. S2†). These two metal ions are coordinated by the nitrogen atoms of **L2**^4–^, featuring a different scenario to that of the Ni cluster in NKU-**112** (Fig. S5†). The ligand in NKU-**113** has two-fold disorder with an occupancy of 0.5 each, and Co2 has four-fold disorder with an occupancy of 0.25 each. There are four kinds of cages in NKU-**113**: the octahedron cage **D**, with a diameter of *ca.* 10.6 Å, is defined by six Co clusters on the vertices and twelve isophthalic moieties in **L2**^4–^; octahedral cage **E**, with a diameter of *ca.* 28.6 Å, is defined by six Co clusters and twelve **L2**^4–^ ligands; distorted cuboctahedral cage **F** features four triangles combined with three Co clusters on the cross-section and six **L2**^4–^ on the edge, and distorted octahedron cage **G** with a diameter of *ca.* 22.0 Å, which has no corresponding structure in NKU-**112**, defined by six Co clusters and six **L2**^4–^ ligands (Fig. 1a and 3b). As for the packing mode of NKU-**113**, each distorted cuboctahedral cage **F** is surrounded by four octahedral **D** cages by sharing three Co clusters and three isophthalic moieties on the surface; it is additionally surrounded by twelve distorted octahedral **G** cages by sharing two Co clusters and half of an **L2**^4–^ ligand on the edge of the cross-section. Half of the **F** cages wrap one octahedral **E** cage, with the other half of the **F** cages wrapping four **G** cages and a **D** cage (Fig. 1d and S7†). Cages **D** and **E** & **F** possess two kinds of pores: a smaller pore in the range of 0.5–0.9 nm in cage **D**, and a larger pore in the range of 1.9–4.5 nm in cage **E** & **F**. Benefiting from the presence of the coordinating bipyridine moiety in the backbone of the **L2**^4–^ ligand, the pyridine group bonds to a different cluster in the framework of NKU-**113**, resulting in the formation of a self-penetrated structure. Accordingly, the tiling structure of the framework of NKU-**113** also changed with respect to that of NKU-**112** because of its self-support property (Fig. S6b†). Topologically, by regarding cage **D** as nodes, the framework of NKU-**113** could be simplified as a 3D binodal (3,18)-connected net with a point symbol of (4^2^·6)_6_(4^60^·6^93^), as determined by the TOPOS software. The solvent-accessible volume of NKU-**113** is estimated by PLATON to be 67.2% per unit cell.

Compared with NKU-**112**, the structure deployment in NKU-**113** causes several changes. The SBU in NKU-**112**, with a formula of [Ni_2_(COO)_4_(μ_2_-H_2_O)(H_2_O)_2_DMF_2_], is similar to that of [Co_2_(COO)_4_(μ_2_-H_2_O)(H_2_O)_2_] in NKU-**113** except for the fact that in the latter cluster the chelating bipyridine moiety in the **L2**^4–^ ligand replaces the coordinating DMF molecules (Fig. S5†). Furthermore, because of the additional coordination of the bipyridine moiety to the metal cluster, NKU-**113** features an additional octahedral cage composed of six **L2**^4–^ ligands and six metal clusters (Fig. 1a) with respect to NKU-**112**. It is known that the coordination of solvent molecules will diminish the rigidity and stability of SBUs, and therefore replacing these coordinated solvent molecules with coordinate bonds between the SBU and the framework can strengthen the overall structure.

In detail, if Co–N bonds in NKU-**113** are ignored, the packing mode of the two frameworks is different from that in NKU-**112**. By regarding cage **A** and cage **D** as nodes and other ligands as linkers, one structure can be simplified into an fcc-like framework (Fig. S8†). Cages **A** & **D** construct tetrahedral and octahedral voids. In NKU-**112**, the second framework occupies the centres of larger octahedral voids. In contrast, the second framework occupies the centers of smaller tetrahedral voids in NKU-**113** (Fig. S9 and S10†). This structure change is induced by the presence of new coordination bonds since the smaller tetrahedral voids are suitable for the linking of the penetrated framework through Co–N bonds. According to the changes in packing mode, the nestification mode in NKU-**113** has also varied (Fig. S11 and S12†). The presence of additional coordinate bonds causes a reduction of the distance between the two structures, which contributes to the increase in porosity and stability observed in NKU-**113** with respect to NKU-**112**. In summary, although NKU-**112** and NKU-**113** have similar building blocks, the insertion of linkers in the latter is associated with obvious differences that lead to an enhancement in the structure stability and porosity.

### Stabilities and adsorption properties of NKU-**112** and NKU-**113**

To evaluate the porosity and gas storage/separation potential of NKU-**112** and NKU-**113**, gas adsorption experiments have been performed. Before the measurements, the samples of NKU-**112** and NKU-**113** were soaked in ethanol for solvent exchange and supercritically dried using carbon dioxide. However, the framework of NKU-**112** partially collapsed after the activation procedures (Fig. 2a), and NKU-**113** showed a well retained framework structure (Fig. 2b). It should be noted that the collapse of NKU-**112** can be ascribed to the loss of coordinated solvents during activation in the SBU as discussed above.

**Fig. 4:**
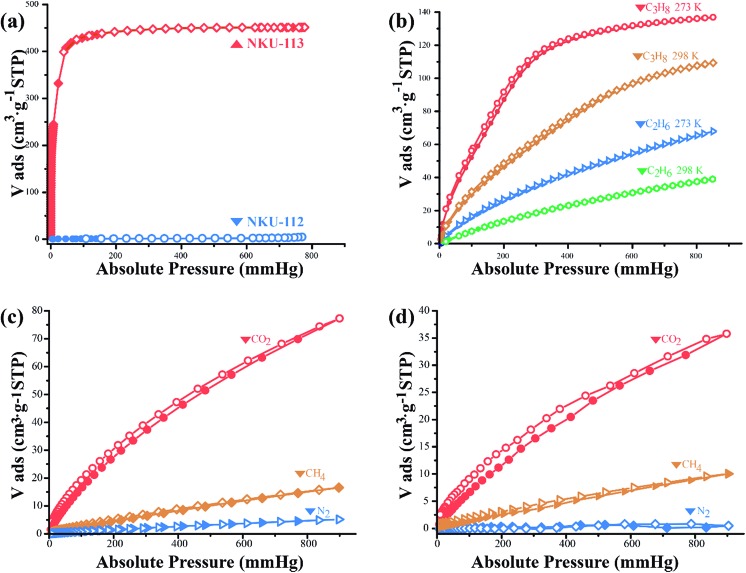
Gas sorption isotherms. (a) N_2_ isotherms of NKU-**112** and NKU-**113** at 77 K. (b) C_3_H_8_ and C_2_H_6_ isotherms of NKU-**113** at 273 K and 298 K. (c and d) CO_2_, CH_4_, and N_2_ isotherms of NKU-**113** at 273 K (c) and at 298 K (d). The filled and open symbols represent the adsorption and desorption data, respectively.

N_2_ sorption tests were first conducted at 77 K to characterize the porosity of the materials (Fig. 4a). The N_2_ adsorption isotherm of NKU-**113** has the typical characteristics of a type I profile, and the Brunauer–Emmett–Teller (BET) surface area and Langmuir surface area are 1486 m^2^ g^–1^ and 1966 m^2^ g^–1^, respectively. The pore distribution analysis performed using the Horvath–Kawazoe (H–K) method shows a main distribution of 0.7–1.2 nm and 1.9–4.2 nm (Fig. S13†), indicating that two kinds of pore existed in the framework, which is consistent with the crystal structures.

Considering the highly porous framework of NKU-**113** and the presence of amide groups which may benefit its gas sorption performance, a series of sorption tests were performed with C_2_H_6_, C_3_H_8_, CO_2_, and CH_4_ (Fig. 4b–d). The gas uptake of NKU-**113** at 273 K is 16 cm^3^ g^–1^ (STP) for CH_4_, 63 cm^3^ g^–1^ (STP) for C_2_H_6_, 135 cm^3^ g^–1^ (STP) for C_3_H_8_ and 77 cm^3^ g^–1^ (STP) for CO_2_. At 298 K, the framework shows gas uptakes of 10 cm^3^ g^–1^ (STP) for CH_4_, 36 cm^3^ g^–1^ (STP) for C_2_H_6_, 105 cm^3^ g^–1^ (STP) for C_3_H_8_, and 36 cm^3^ g^–1^ (STP) for CO_2_. On the basis of the investigation of capacities, the heat of sorption of different gases was also investigated (Fig. S14†). The initial heat of sorption values are 15.4 kJ mol^–1^ for CH_4_, 28.2 kJ mol^–1^ for C_2_H_6_, 27.2 kJ mol^–1^ for C_3_H_8_ and 30.4 kJ mol^–1^ for CO_2_. The considerable uptakes and heat of adsorption values of NKU-**113** towards alkanes and CO_2_ suggest its potential in gas storage and separation applications.

## Conclusions

In summary, through the tuning of coordination sites in organic ligands, interpenetrated MOF NKU-**112** and self-penetrated MOF NKU-**113** have been constructed. Owing to the additional chelating bipyridine coordination sites introduced through the ligand, the NKU-**113**, featuring a self-penetrated framework, reveals enhanced stability and porosity compared with those of the interpenetrated framework of NKU-**112**. The approach reported herein may provide a valuable method for the stability and gas sorption performance enhancement of penetrated MOFs.

## Conflicts of interest

There are no conflicts to declare.

## Supplementary Material

Supplementary information

Crystal structure data
