# Supplementary material for: Enhancing the stability and porosity of penetrated metal–organic frameworks through the insertion of coordination sites
Source: Chem Sci. 2017 Nov 15;9(4):950–5. doi: 10.1039/c7sc04192f (PMC5873222; doi:10.1039/c7sc04192f)
Supplement: Supplementary file 1 [file SC-009-C7SC04192F-s001.pdf]

Electronic Supplementary Material (ESI) for Chemical Science.
This journal is © The Royal Society of Chemistry 2017

Electronic Supplementary Information (ESI)

Enhancing stability and porosity of penetrated metal–organic frameworks through insertion of coordination sites

Rui Feng,‡[a] Yan-Yuan Jia,‡[a] Zhao-Yang Li,[b] Ze Chang[b] and Xian-He Bu*[ab]

[a]State Key Laboratory of Elemento-Organic Chemistry, College of Chemistry, Collaborative Innovation Center of Chemical Science and Engineering (Tianjin), Nankai University, Tianjin 300071, China.

[b]School of Materials Science and Engineering, National Institute for Advanced Materials, Tianjin Key Laboratory of Metal and Molecule-Based Material Chemistry, Nankai University, Tianjin 300350, China.

E-mail: buxh@nankai.edu.cn. Fax: +86-22-23502458.

‡ Authors R. Feng and Y.-Y. Jia contributed equally to this work.

Materials and Methods

The H_4**L1** and H_4**L2** ligands were synthesized according to procedures from the reported literatures. [S1-S2] All the chemicals were purchased from commercial sources and used without further purification. Powder X-ray diffraction (PXRD) patterns were recorded with a Rigaku D/Max-2500 diffractometer at 40 kV and 100 mA for a Cu-target tube and a graphite monochromator. Thermogravimetric analyses (TGA) were carried out on a Rigaku standard TG-DTA analyzer with a heating rate of 10 °C·min^{-1}, using an empty Al_2O_3 crucible as reference. Infrared analyses (IR) spectra were measured on a Bruker TENSOR 37 FT-IR Spectroscopy. The simulated PXRD pattern was obtained based on the single-crystal data by diffraction crystal module of the Mercury (Hg) program version 1.4.2 available free of charge via the Internet at http://www.iucr.org/.

Crystal Structure Determination

All diffraction data were collected on a Rigaku SCX-mini diffractometer at 293(2) K with Mo-Kα radiation (λ = 0.71073 Å) by ω scan mode. The structures were solved by direct methods using the SHELXS program of the SHELXTL package and refined with SHELXL[S3]. The disordered solvent molecules NKU-**112** and NKU-**113** were removed by SQUEEZE as implemented in PLATON[S4] and the results were appended in the CIF files.

Synthesis of NKU-112

NKU-**112** ($[Ni_2$**L1**$(\mu_2\text{-}H_2O)(H_2O)_2(DMF)_2]\cdot(\text{solvents})_n$) was synthesized by the solvothermal reaction of H_4**L1** (0.21 mmol) and $Ni(NO_3)_2\cdot 6H_2O$ (0.07 mmol) in N,N-Dimethylformamide (DMF, 3 mL), acetonitrile (CH_3CN, 1 mL) and H_2O (1 mL) at 75°C for 72 hours to give green block crystals (Yield: ~56% based on H_4**L1**). IR (KBr, cm^{-1}): 3425s, 2093w, 1657s, 1522s, 1423m, 1375s, 1326m, 1280m, 1149m, 1103m, 912w, 860w, 782s, 721s, 665m, 601m.

Synthesis of NKU-113

NKU-**113** ($[Co_2$**L2**$(\mu_2\text{-}H_2O)(H_2O)_2]\cdot(\text{solvents})_n$) was synthesized by the solvothermal reaction of H_4**L2** (0.21 mmol) and $Co(NO_3)_2\cdot 6H_2O$ (0.07 mmol) in N,N-Dimethylformamide (DMF, 3 mL), acetonitrile (CH_3CN, 1 mL) and H_2O (1 mL) at 75°C for 72 hours to give red block crystals (Yield: ~45% based on H_4**L2**). IR (KBr,

cm^{-1}): 3451s, 2308w, 1657s, 1555s, 1425m, 1376s, 1289m, 1150w, 1105w, 1039w, 783m, 717s, 670m, 629m, 602m.

Adsorption Measurements

Gas adsorption measurements were performed using an ASAP 2020M gas adsorption analyzer. Before the measurements, the supercritical dried samples were activated under high vacuum (less than 10^{-5} Torr) at 150 °C. About 80 mg activated samples were used for gas sorption measurements. Isotherms were collected at 77 K with a liquid nitrogen bath, at 273 K and with an ice water mixture bath, and at 298 K in an electric heating jacket.

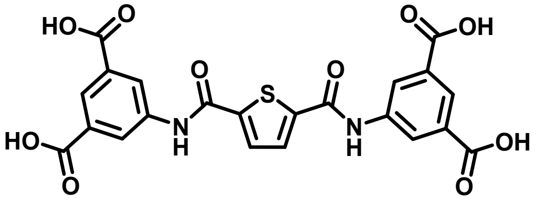

5,5'-((thiophene-2,5-dicarbonyl)bis(azanediyl))diisophthalic acid

H_4L1

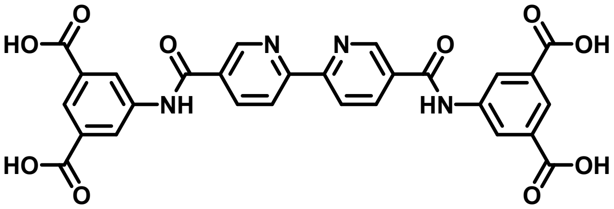

5,5'-(([2,2'-bipyridine]-5,5'-dicarbonyl)bis(azanediyl))diisophthalic acid

H_4L2

Figure S1. The structures of H_4**L1** and H_4**L2**.

Table S1. The crystallography data of NKU-**112** and NKU-**113**.

	**NKU-112**	**NKU-113**
Formula	$C_{28}H_{30}N_4Ni_2O_{15}S$	$C_{28}H_{20}Co_2N_4O_{13}$
Fw	812.00	738.35
Space group	*Ia*-3	*Fd*-3*m*
a (Å)	39.7584(2)	46.6983(3)
b (Å)	39.7584(2)	46.6983(3)
c (Å)	39.7584(2)	46.6983(3)
α (deg)	90	90
β (deg)	90	90
γ (deg)	90	90
V (Å^3)	62847.3(9)	101836.4(11)
Z	48	48
D (g/cm^{-3})	1.030	0.700
μ (mm^{-1})	1.702	3.377
T (K)	293(2)	293(2)
R [a)]/$wR2$ [b)]	0.0721/0.1988	0.1495/0.3603
Completeness	99.8 %	96.2%
GOF on F^2	1.022	1.059
CCDC number	1576271	1576272

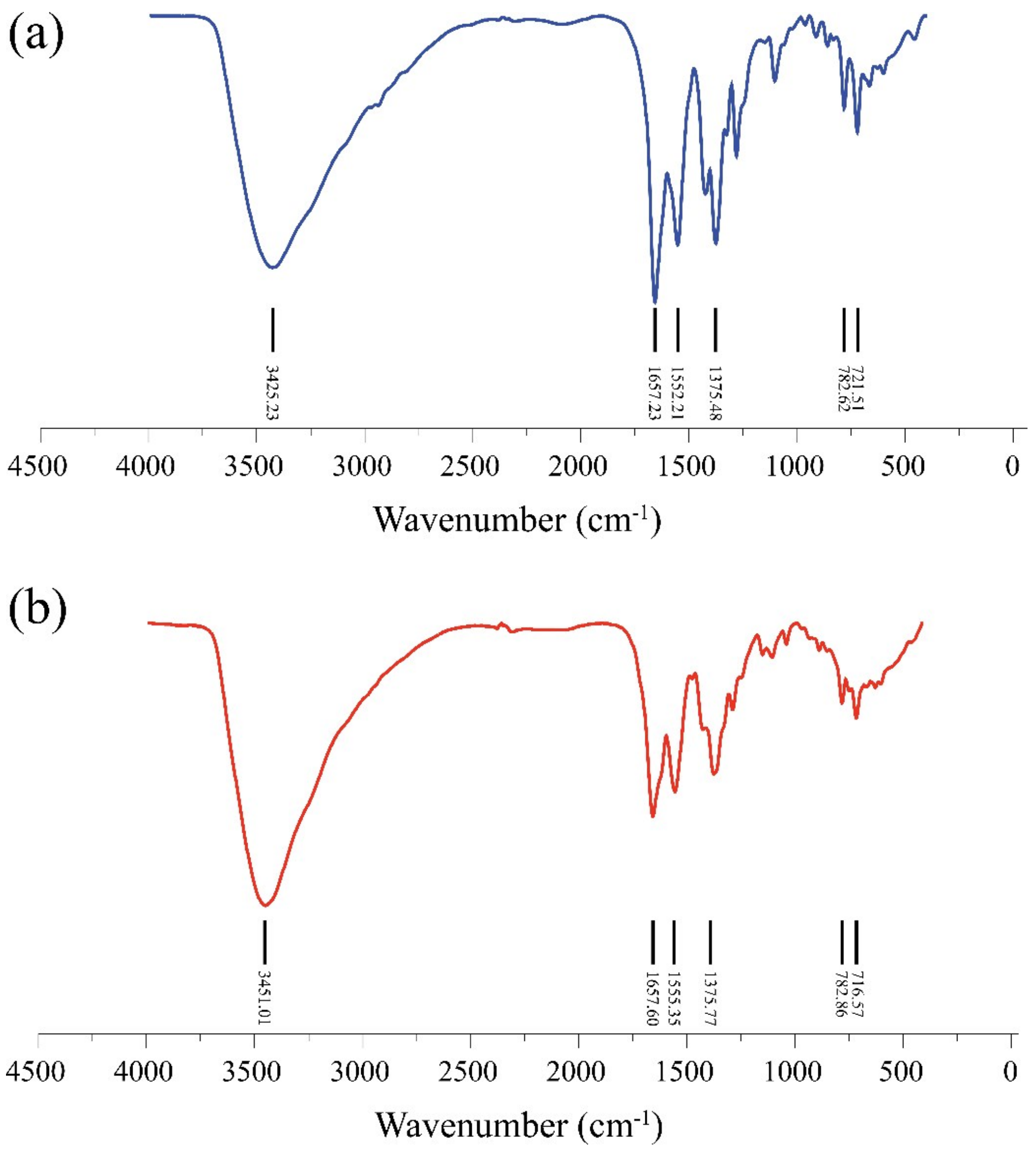

Figure S2. The IR spectra of NKU-**112** (a) and NKU-**113** (b).

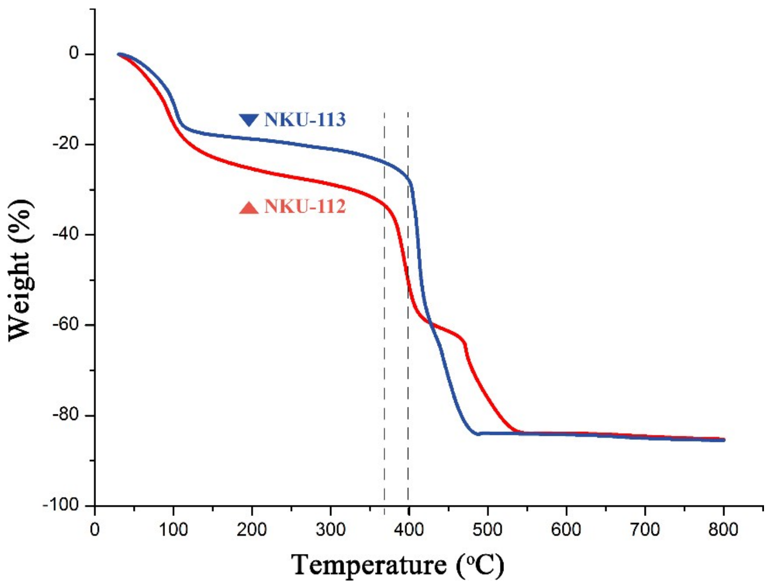

Figure S3. The TG profiles of NKU-**112** (red) and NKU-**113** (blue).

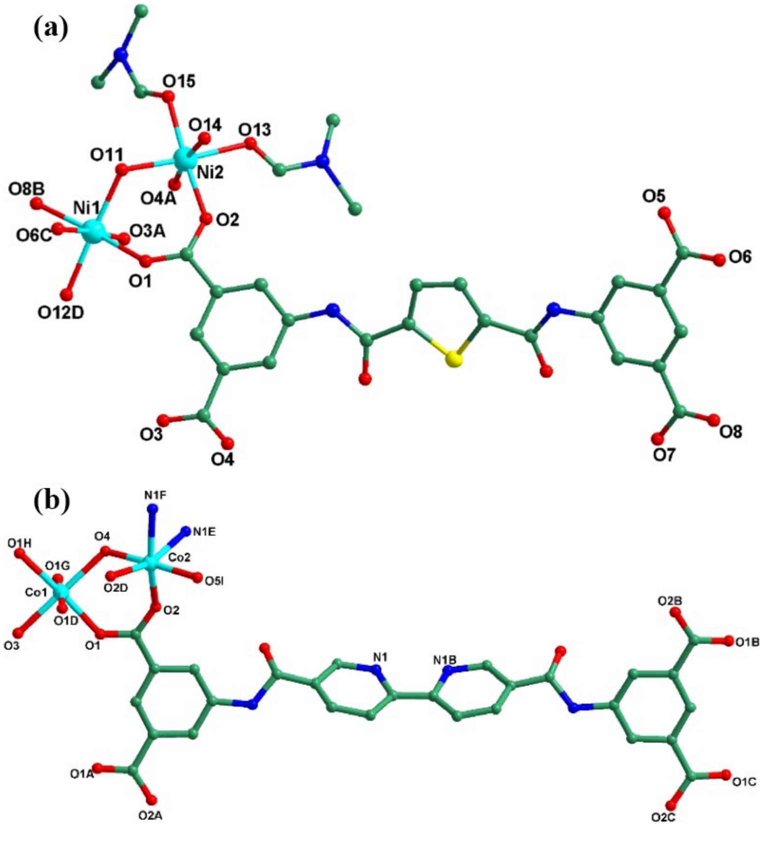

Figure S4. The coordination environment diagrams of NKU-**112** (a) and NKU-**113** (b).

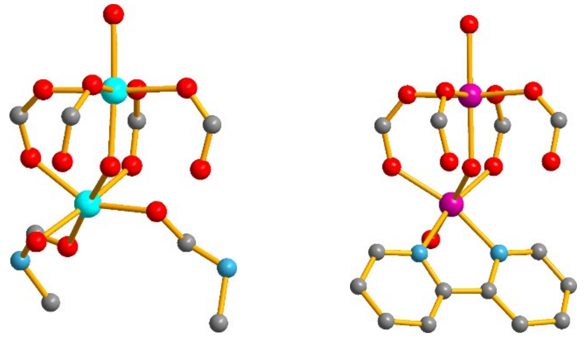

Figure S5. The structure of SBU in NKU-**112** (left) and NKU-**113** (right).

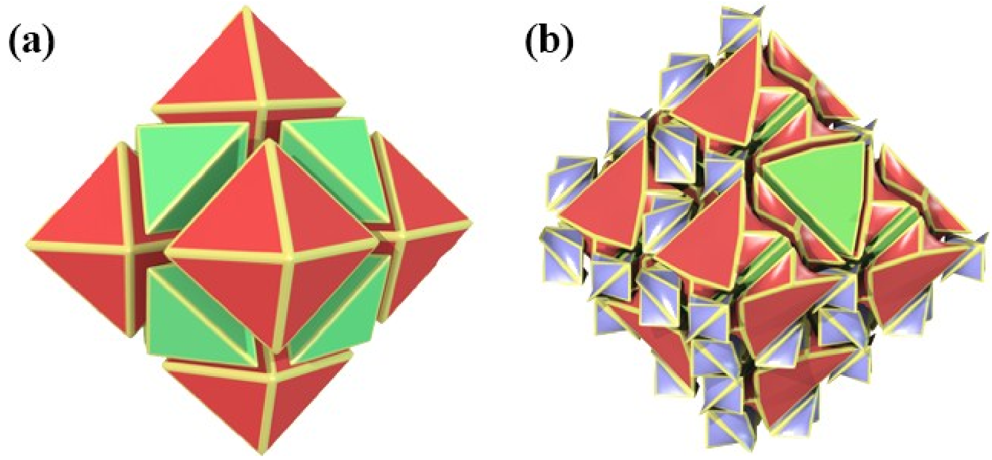

Figure S6. The tilling diagrams of NKU-**112** (a) and NKU-**113** (b).

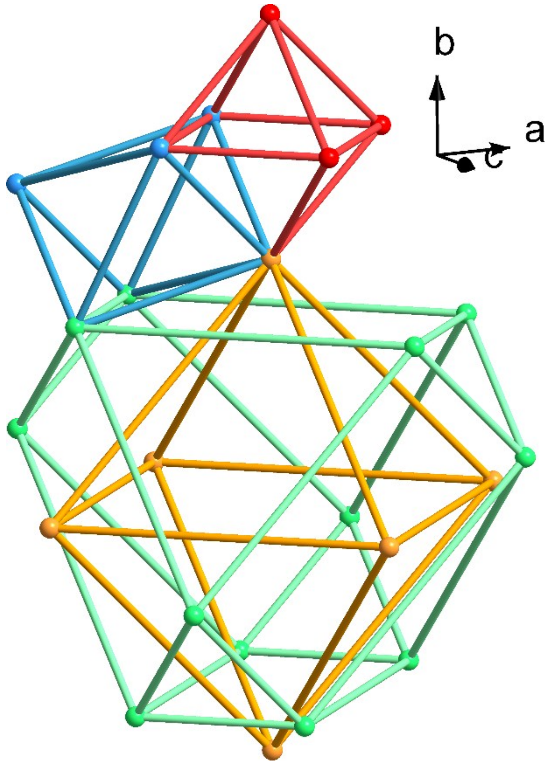

Figure S7. Diagram of the position relationships of cages in NKU-**113**, cage **E (yellow)** is wrapped by cage **F (green)**.

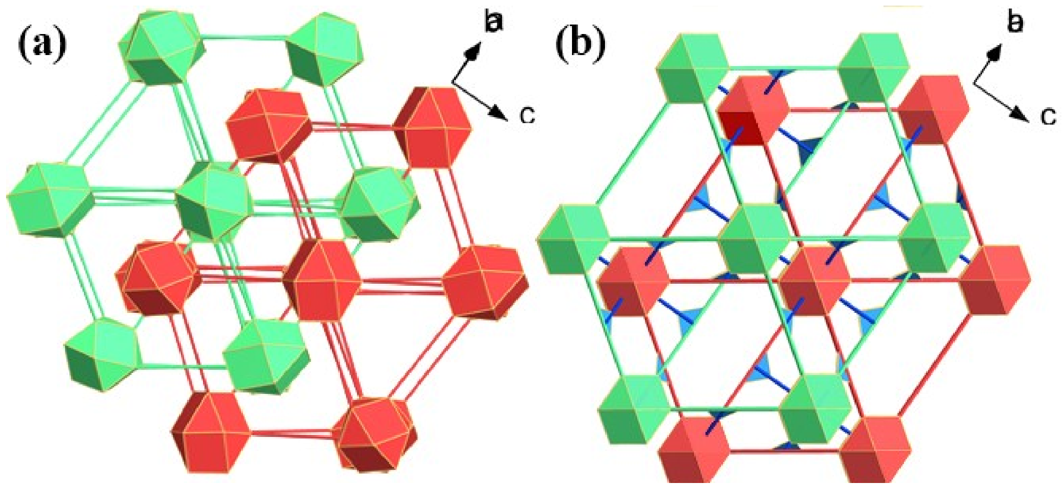

Figure S8. Diagrams of the interpenetrated framework of NKU-**112** (a) and the self-penetrated framework of NKU-**113** (b).

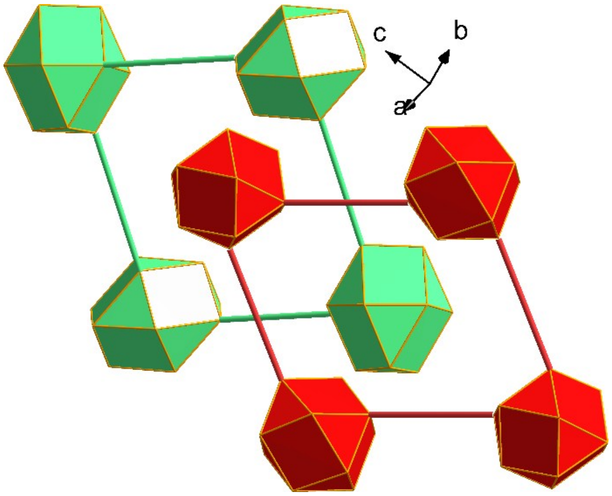

Figure S9. The interpenetrated cages in NKU-**112**.

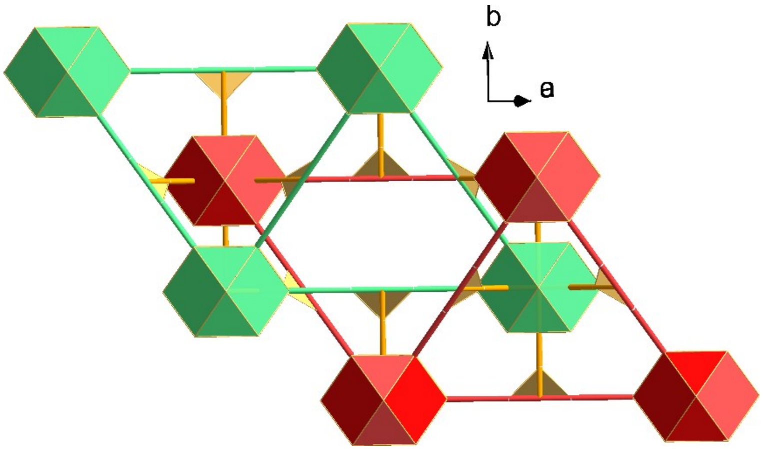

Figure S10. The self-penetrated cages in NKU-**113**.

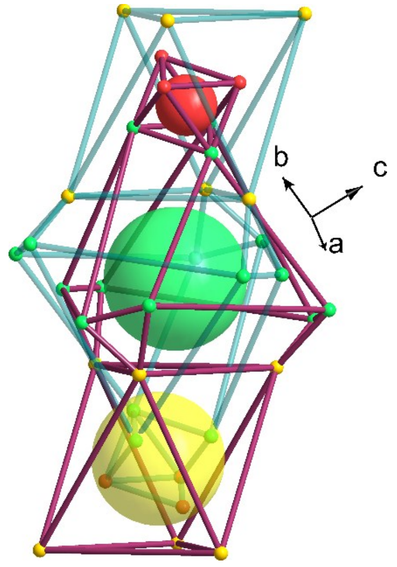

Figure S11. Diagram of the interpenetrated two sets of three cages in NKU-**112**.

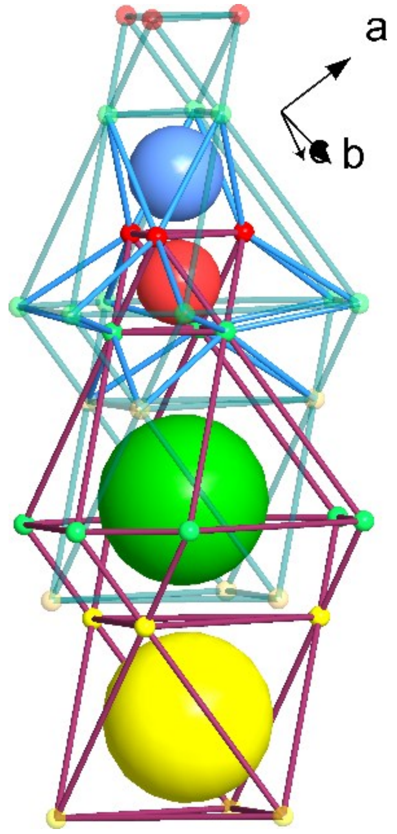

Figure S12. Diagram of the interpenetrated two sets of three cages in NKU-**113**.

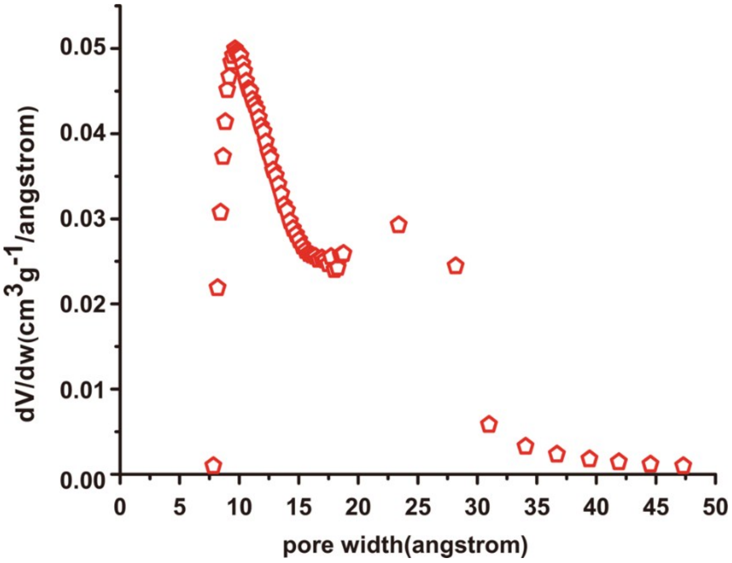

Figure S13. Pore size distribution plot of NKU-**113**.

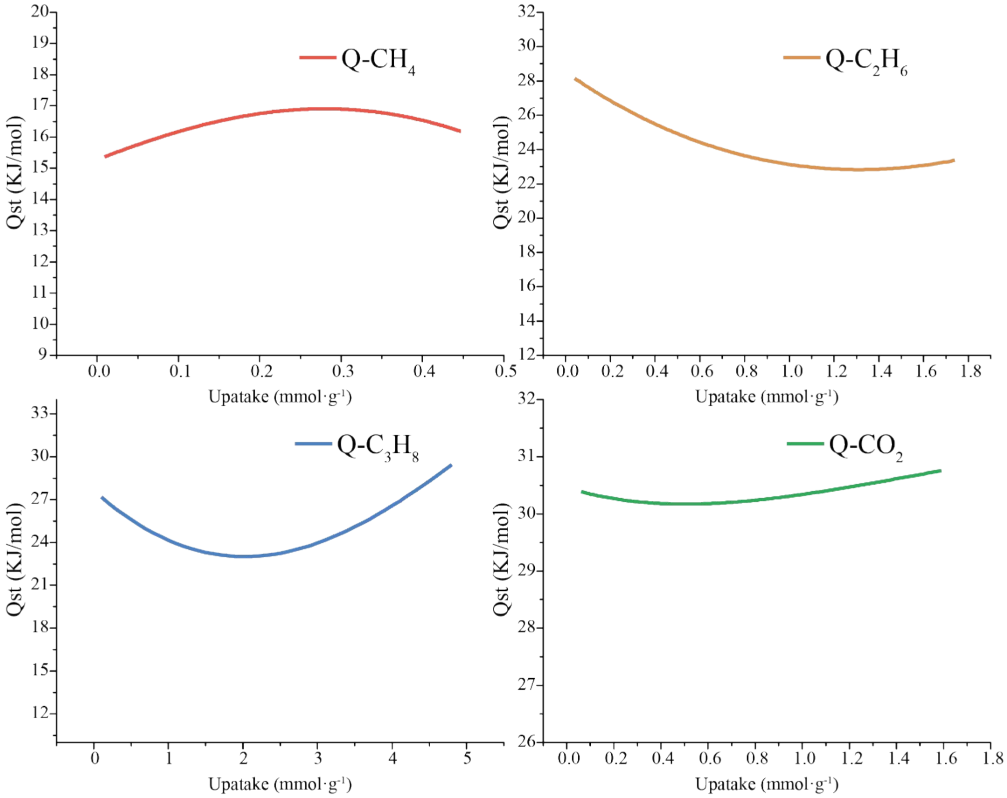

Figure S14. The heat of adsorption of CH_4, C_2H_6, C_3H_8, and CO_2 in NKU-**113**.

References

[S1] T. T. Wang, Y. Y. Jia, Q. Chen, R. Feng, S. Y. Tian, T.-L. Hu, X.-H. Bu. *Sci. China Chem.* **2016**, *59*, 959-964.

[S2] X.-T. Liu, Y.-Y. Jia, Y.-H. Zhang, G.-J. Ren, R. Feng, S.-Y. Zhang, M. J. Zaworotko, X.-H. Bu, *Inorg. Chem. Front.* **2016**, *3*, 1510-1515.

[S3] G. M. Sheldrick, SHELXL97, *Program for Crystal Structure Refinement*; University of Göttingen: Göttingen, Germany, 1997.

[S4] A. L. Spek, *J. Appl. Crystallogr.* **2003**, *36*, 7.
